# Data on Arc and Zif268 expression in the brain of the α-2A adrenergic receptor knockout mouse

**DOI:** 10.1016/j.dib.2016.02.007

**Published:** 2016-02-10

**Authors:** Jeff Sanders

**Affiliations:** Department of Pharmacology and Experimental Neuroscience, 985800 Nebraska Medical Center, Omaha, NE 68198-5800, USA

**Keywords:** Arc, Activity Regulated Cytoskeleton Associated Protein, PBS, phosphate-buffered saline, α2A-AR KO, alpha-2A adrenergic receptor knockout, WT, wild-type, i.p, intraperitoneal, hr, hour, mRNA, messenger ribonucleic acid, Norepinephrine, Arc, Zif268, Adrenergic, Cortex, Hippocampus

## Abstract

The α2-adrenergic receptor (α2-AR) is widely distributed in the brain with distinct roles for α2-AR subtypes (A, B and C). In this article, data are provided on Activity Regulated Cytoskeleton Associated Protein (Arc) and Zif268 expression in the brain of the α2A-AR knockout (α2A-AR KO) mouse. These data are supplemental to an original research article examining Arc and Zif268 expression in rats injected with the α2-AR antagonist, RX821002 (http://dx.doi.org/10.1016/j.neulet.2015.12.002. [1]).

**Specifications table**

**Table:** 

Subject area	Biology
More specific subject area	Neuropharmacology
Type of data	Image, figure, graph
How data was acquired	*in situ* hybridization to Arc mRNA and Zif268 mRNA.
Data format	Autoradiography processed with image analysis
Experimental factors	α2A-AR KO KO and WT mice were injected i.p. with saline.
Experimental features	1 h after treatments, brains were harvested and then analyzed for Arc and Zif268 mRNA.
Data source location	Department of Pharmacology and Experimental Therapeutics. University of Nebraska Medical Center, Omaha, Nebraska.
Data accessibility	Data are available with this article.

**Value of the data**

• These data may stimulate research into the role of specific α2-AR subtypes in regulating cortical and hippocampal plasticity.
• These data may stimulate research into the brain activity of α2A-AR KO mice in behavioral models of stress and anxiety [2].
• These data may stimulate research into learning and memory processes of mice with deletions of α2-AR subtypes.

## 1 Data

**Fig. 1:**
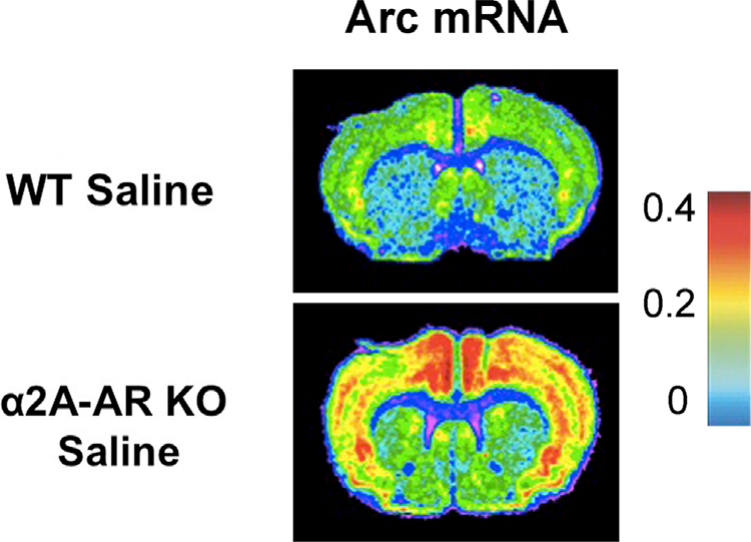
Representative Arc mRNA *in situ* hybridization autoradiographs. Arc mRNA in the brains of saline injected WT and α2A-AR KO mice. The calibration bar indicates the density of Arc mRNA and is calibrated in μCi/mg tissue.

**Fig. 2:**
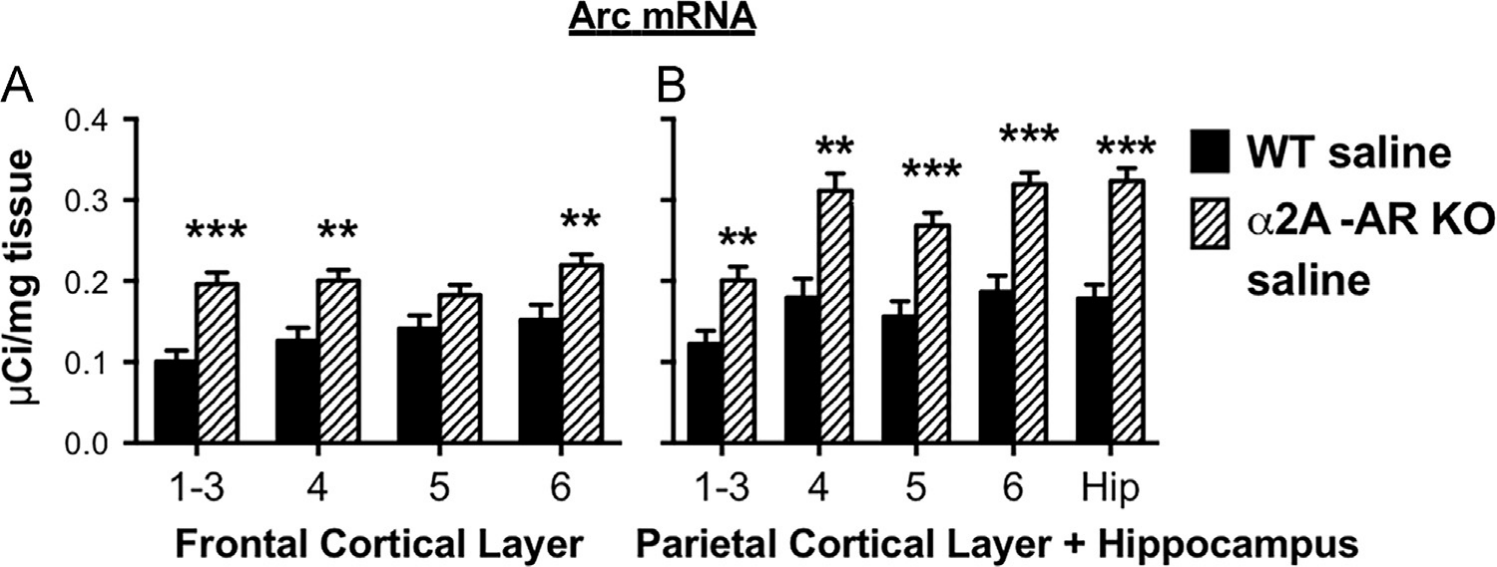
Arc mRNA in cortex and hippocampus of saline injected WT and α2A-AR KO mice. (A) Arc mRNA in frontal cortex of saline injected WT and α2A-AR KO mice. (B) Arc mRNA in the parietal cortex and hippocampus of saline injected WT and α2A-AR KO mice. Number refers to cortical layer. WT=wild type mouse, α2A-AR KO= alpha-2A adrenergic receptor knockout mouse, Hip=Hippocampus. **p<0.01, ***p<0.001.

**Fig. 3:**
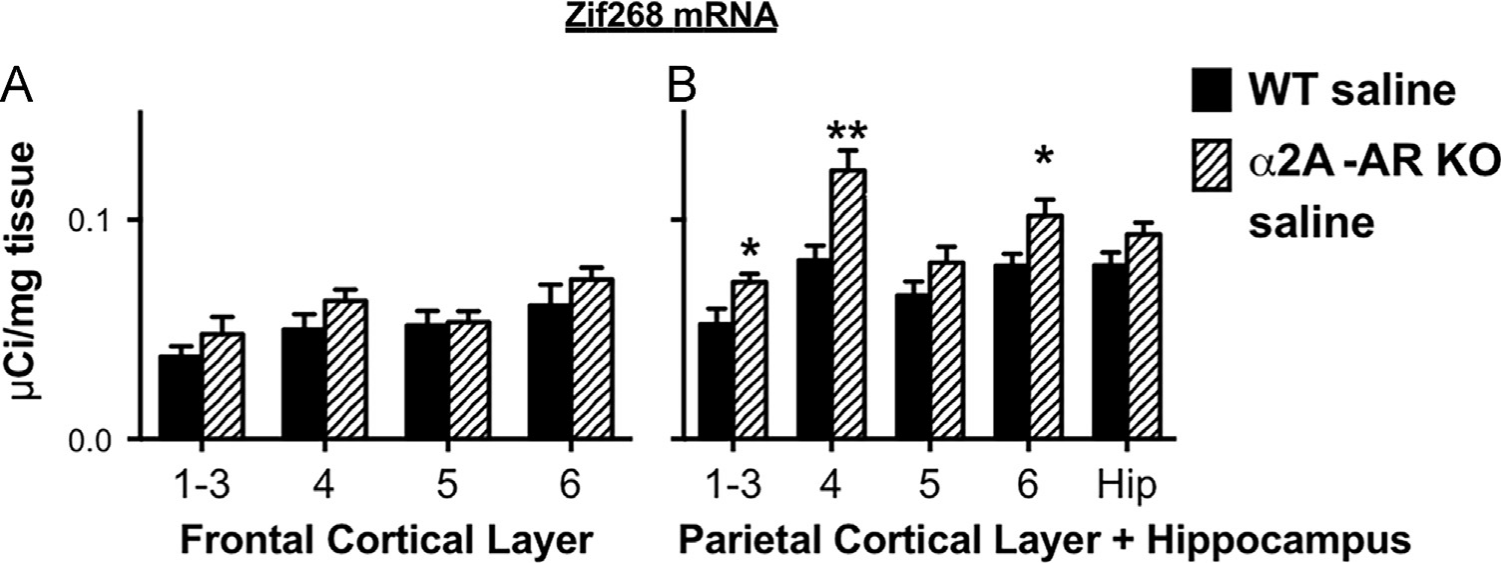
Zif268 mRNA in cortex and hippocampus of saline injected WT and α2A-AR KO mice. (A) Zif268 mRNA in frontal cortex of saline injected WT and α2A-AR KO mice. (B) Zif268 mRNA in parietal cortex and hippocampus of saline injected WT and α2A-AR KO mice. Number refers to cortical layer. WT=wild type mouse, α2A-AR KO= alpha-2A adrenergic receptor knockout mouse, Hip=Hippocampus. *p<0.05, **p<0.01.

These data show Arc and Zif268 mRNA levels in the brains of saline injected WT mice compared to saline injected α2A-AR KO mice. (Fig. 1, Fig. 2, Fig. 3).

## 2 Experimental design, materials and methods

Male C57 Bl/6J mice were purchased from Charles River Laboratories (Wilmington, MA), and are designated as wild type (WT) mice in this data report. Male α2A-AR KO mice were purchased from Jackson Labs. WT and α2A-AR KO mice (*n*=5 per group) were injected i.p. with 100 μL of saline. Brains were harvested 1 h later, frozen on dry ice and stored at −80 °C. All animal use procedures were in strict accordance with The National Institutes of Health Guide for the Care and Use of Laboratory Animals and were approved by the University of Nebraska Medical Center Animal Care and Use Committee.

### 2.1 in situ hybridization

*in situ* hybridization to Arc and Zif268 mRNA was performed as previously described [1], [3]. Briefly, sixteen-micron tissue sections were cut in a cryostat and thaw-mounted on Superfrost Plus slides (Fisher Scientific, Pittsburgh, PA). Sections were fixed in ice cold 4% paraformaldehyde and hybridized with oligonucleotide probe sequences to Arc mRNA (5′-CTT-GGT-TGC-CCA-TCC-TCA-CCT-GGC-ACC-CAA-GAC-TGG-TAT-TGC-TGA-3′) and Zif268 mRNA (5′-CCG-TTG-CTC-AGC-AGC-ATC-ATC-TCC-TCC-AGT-TTG-GGG-TAG-TTG-TCC-3′). A Blast search of Genbank found that these sequences did not have significant homology with other sequences. Probes were 3′ end labeled with [^35^S]-dATP (1200 Ci/mmol, Perkin Elmer, Boston, MA) using terminal deoxyribonucleotidyl transferase (3′ End Labeling System, Perkin Elmer). Hybridization buffer containing 1×10^6^ cpm of labeled probe was applied to each slide. Slides were coverslipped, sealed with D.P.X. (Aldrich Chemical Co., Milwaukee, WI) and placed overnight in a 1XSSC humidified sealed Tupperware container at 42 °C. The next day coverslips were removed in 55 °C 1XSSC and slides were washed 4×15 min in 1XSSC at 55 °C. Slides were apposed to Biomax film (Kodak, Rochester, NY) for 2–3 weeks. Films were developed using standard techniques and analyzed using the MCID-M7 image analysis system (Interfocus Imaging, Ltd., Linton, England).

### 2.2 Image analysis

Arc and Zif268 mRNA levels in saline injected WT and α2A-AR KO mice were quantified with image analysis. Autoradiographic densities were quantified using commercial tritium standards (American Radiochemicals, St. Louis, MO) that were previously calibrated to ^35^S [4]. Expression in mice was measured at two coronal levels. These levels corresponded to 0.86 mm anterior to the bregma and 1.70 mm posterior to the bregma, and referred to as frontal and parietal cortex, respectively.

## 3 Statistics

Arc and Zif268 mRNA levels were compared in saline injected WT and α2A-AR KO mice with a Student׳s *t*-test in each cortical layer and in the hippocampus.
