# Supplementary material for: Data on Arc and Zif268 expression in the brain of the α-2A adrenergic receptor knockout mouse
Source: Data Brief. 2016 Feb 10;7:8–11. doi: 10.1016/j.dib.2016.02.007 (PMC4761656; doi:10.1016/j.dib.2016.02.007)
Supplement: Supplementary file 1 — Supplementary material [file mmc1.docx]

**Disclosures:** The author declares no conflict of interest, financial or otherwise. Dr Sanders performed all experiments, analyzed the data and wrote the manuscript. Dr Sanders approves the final article.
